# Gender differences in non-motor symptoms in Parkinson’s disease: a multicenter longitudinal study

**DOI:** 10.1007/s10072-026-09031-2

**Published:** 2026-04-16

**Authors:** Sofia Cuoco, Michela Russo, Marina Picillo, Carlo Ricciardi, Maria Claudia Russillo, Marianna Amboni, Gennarina Arabia, Laura Avanzino, Margherita Canesi, Alessia Catania, Roberto Ceravolo, Calogero Edoardo Cicero, Roberto Cilia, Isabel Colangelo, Giovanna Dati, Rosa De Micco, Anna De Rosa, Alessio Di Fonzo, Roberto Eleopra, Vincenza Fetoni, Barbara Garavaglia, Augusta Giglio, Martina Giuntini, Federica Invernizzi, Giulia Lazzeri, Roberta Marchese, Alessandra Nicoletti, Claudio Pacchetti, Celeste Panteghini, Manuela Pilleri, Fabiana Giada Radicati, Chiara Reale, Cesa Scaglione, Andrea Soricelli, Fabrizio Stocchi, Alessandro Tessitore, Laura Vacca, Maria Antonietta Volontè, Graziella Volpi, Roberta Zangaglia, Francesco Amato, Paolo Barone, Maria Teresa Pellecchia

**Affiliations:** 1 Department of Medicine, Surgery and Dentistry Scuola Medica Salernitana, Neuroscience Section, University of Salerno, Via Allende, 84081 Salerno, Baronissi Italy; 2 Department of Chemical, Material and Industrial Production Engineering, University of Naples FEDERICO II, Naples, Italy; 3 Department of Electrical Engineering and Information Technology, University of Naples FEDERICO II, Naples, Italy; 4 SC Neurologia E Stroke Unit, Ospedale Santa Croce Moncalieri, Turin, Italy; 5 Department of Medical and Surgical Sciences, Institute of Neurology, Magna Græcia University, Catanzaro, Italy; 6 Department of Experimental Medicine (DIMES), Section of Human Physiology, University of Genoa, Genoa, Italy; 7 IRCCS Azienda Ospedale Metropolitana, Genoa, Italy; 8 Neurorehabilitation Unit, Parkinson’s Disease and Movement Disorders Center, Moriggia-Pelascini Hospital, Gravedona Ed Uniti, Como Italy; 9 Medical Genetics and Neurogenetics Unit, Fondazione IRCCS Istituto Neurologico “C. Besta”, Milan, Italy; 10 Department of Clinical and Experimental Medicine, University of Pisa, Pisa, Italy; 11 Neurologic Unit, AOU “Policlinico-San Marco”, Department of Medical, Surgical Sciences and Advanced Technologies, GF Ingrassia, University of Catania, Catania, Italy; 12 Department of Clinical Neurosciences, Parkinson and Movement Disorders Unit, Fondazione IRCCS Istituto Neurologico Carlo Besta, Milan, Italy; 13 Department of Advanced Medical and Surgical Sciences, University of Campania “Luigi Vanvitelli”, Naples, Italy; 14 Department of Neurosciences and Reproductive and Odontostomatological Sciences, Federico II University, Naples, Italy; 15 IRCCS Ca’ Granda Ospedale Maggiore Policlinico, Neurology Unit, Milan, Italy; 16 UOS Neurologia P.O. Fatebenefratelli-ASST Fatebenefratelli-Sacco, Milan, Italy; 17 Centro Parkinson E Parkinsonismi, Milan, Italy; 18 Parkinson’s Disease and Movement Disorders Unit, IRCCS Mondino Foundation, Pavia, Italy; 19 IRCCS San Raffaele, Rome, Italy; 20 IRCCS Istituto Delle Scienze Neurologiche Di Bologna, Bologna, Italy; 21 IRCCS SYNLAB SDN, Naples, Italy; 22 University San Raffaele, Rome, Italy; 23 IRCCS San Raffaele Scientific Institute, Neurology Unit, Milan, Italy; 24 SSD Malattie Endocrine Diabetologia, ASST Fatebenefratelli-Sacco, Milan, Italy

**Keywords:** Early-stage Parkinson’s disease, Levodopa-naïve patients, Longitudinal study, Non-motor disorders, Sex differences

## Abstract

**Background:**

Non-motor symptoms (NMSs) are highly prevalent in Parkinson’s disease (PD) and affect patients’ quality of life. Data on gender differences in NMSs are largely cross-sectional and derived from chronically treated populations. Longitudinal evidence in early, levodopa-naïve PD patients remains limited. This study aims to longitudinally investigate gender differences in a wide range of NMSs in early-stage levodopa-naïve PD patients during the first 2 years following levodopa initiation.

**Methods:**

This multicenter, prospective study enrolled 216 levodopa-naïve PD patients (139 men, 77 women) from 17 Italian movement disorder centers. Patients were evaluated at baseline and after 24 months (24 M) using validated scales. Baseline gender differences were explored using group comparisons. Gender effects at 24 M were examined using ANCOVA models adjusted for baseline values and levodopa dose at follow-up.

**Results:**

At baseline, women showed greater cardiovascular and thermoregulatory autonomic dysfunction, higher anxiety, pain, fatigue, and worse quality of life, whereas men exhibited greater sexual dysfunction, daytime sleepiness, and better attentional performance. At the 24 M, gender differences persisted only for anxiety, pain, mobility, and emotional well-being, while additional significant differences emerged, including hypersexuality, visuo- spatial domain, and orthostatic-hypotension. Women exhibited greater symptom severity than men across all aforementioned variables, with the exception of hypersexuality.

**Conclusions:**

Gender significantly influences the expression and early evolution of NMSs in PD, independently of levodopa exposure. Gender-specific NMSs profiles are already evident in the first two years of treatment. These findings highlight the importance of integrating gender considerations into early assessment and personalized management of NMSs in PD.

**Supplementary Information:**

The online version contains supplementary material available at 10.1007/s10072-026-09031-2.

## Introduction

Parkinson’s disease (PD) is a neurodegenerative disease characterized by a wide range of motor and non-motor symptoms (NMSs). NMSs include autonomic symptoms, such as cardiovascular, gastrointestinal, urinary, sexual and thermoregulatory features, cognitive deficits, behavioral symptoms, such as depression, apathy, anxiety, hallucinations, impulse control disorders, sleep disturbances and others, such as pain, smell dysfunction and fatigue [1].

NMSs affect over 90% of people with PD at all stages and have a significant impact on them, adding substantial burden to patients' lives. Nevertheless, NMSs are frequently underdiagnosed and inadequately treated [2].

The recent PD literature has underlined gender differences in prevalence, characteristics and managing of NMSs [3]. Gender differences are possibly due to genetic factors, which might differentially influence the non-motor manifestations, estrogens, which appear to exert a protective effect in women [4], but also with neuroendocrine, biochemical and clinical factors [5].

Data regarding NMSs in PD are heterogeneous due to different study design and characteristics of the samples examined [6] (Russillo et al., 2022). Generally, diurnal somnolence, urinary and sexual dysfunction, and cognitive problems are more prevalent in men than women [7–11] Also, behavioral symptoms and REM sleep behavior disorder occur earlier and more often in men than women [12]. On the other hand, women seem to have a greater prevalence of non-motor fluctuations, fatigue, depression, anxiety, and sleep problems than men [7–11].

Despite a growing interest in the role of gender in PD, there remains an unmet need in research and routine clinical practice [13]. Moreover, most studies examining gender differences in non-motor symptoms are cross-sectional and conducted in patients chronically treated with dopaminergic drugs.

In our study, we aim to longitudinally investigate gender differences in NMSs in a sample of early levodopa-naïve PD patients followed for 2 years after starting levodopa treatment.

## Materials and methods

### Study population

This study was a longitudinal, prospective, multicenter investigation carried out across 17 Italian centers specialized in movement disorders. At baseline, levodopa-naïve patients diagnosed with idiopathic PD, either drug-naive or already receiving other antiparkinsonian treatment, were enrolled before their first-ever levodopa intake and followed up for two years after starting levodopa treatment.

Enrollment was conducted as part of routine medical practice. Evaluations were performed at baseline and after a 24-month (24 M) follow-up.

#### Inclusion criteria

Patients were included in the study if they fulfilled the following inclusion criteria:

- PD diagnosis according to the MDS clinical diagnostic criteria [14]
- Needing to start Levodopa therapy as per clinical judgement
- aNever treated with dopaminergic therapy or under treatment with i-MAO (e.g. selegiline, rasagiline) and/or dopamine agonist (pramipexole or ropinirole or rotigotine)
- Age ranging from 41 to 85-years at screening
- Capable of performing all the evaluations at baseline and at the following visits, as judged by the clinician
- Able to provide the informed consent
- Patient should be neither resident nor planned to be admitted to a nursing home at screening
- aUnder stable treatment with other medications for at least 30 days prior to the screening visit

#### Exclusion criteria

Patients were excluded from the study if met any of the following criteria:

- Any among the following: a) acute onset of neurologic symptoms; b) head trauma at the onset of neurologic symptoms; c) cerebellar ataxia; d) early and severe loss of independence and/or postural instability and/or falls
- Neurologic or psychiatric comorbidity
- Active suicidal ideation
- Diagnosis of dementia according to DSM IV
- Concomitant treatment with memantine, acetylcholinesterase inhibitors, antipsychotics (except for low doses of quetiapine) and mood stabilizer (valproate, lithium)
- Treatment with deep brain stimulation for PD
- Presence of relevant clinical conditions that could interfere with the patient’s ability to participate in the study, put the patient at risk or undermine the interpretation of the results
- Brain imaging findings suggesting alternative diagnosis to idiopathic PD; neuroimaging was not required for the study, however it is commonly performed during the diagnostic process as per clinical practice
- aKnown hypersensitivity to levodopa.

### Non-motor symptoms assessments

NMSs were assessed using a comprehensive set of clinical scales specifically designed or validated for NMSs assessment in PD. Behavioral complications were evaluated with the Questionnaire for Impulsive-Compulsive Disorders in Parkinson’s Disease (QUIP) [S1, supplemental material], the Hamilton Depression Rating Scale (HAM-D) [S2, supplemental material], the Hamilton Anxiety Rating Scale (HAM-A) [S3, supplemental material], and the Apathy Evaluation Scale (AES) [S4, supplemental material]. General non-motor symptoms were measured using the MDS-UPDRS non-motor section (Part I) and the Non-Motor Symptoms Severity Scale (NMSS) [S5, supplemental material]. Autonomic symptoms were assessed with the Scale for Outcomes in Parkinson’s Disease—Autonomic (SCOPA-AUT) [S6, supplemental material]. Global cognitive function was evaluated using the Montreal Cognitive Assessment (MoCA) [S7, supplemental material], while quality of life was measured with the 39-item Parkinson’s Disease Quality of Life questionnaire (PDQ-39) [S8, supplemental material]. Subscore/subdomain analyses were performed according to different scale structure.

### Statistical analysis

A statistical analysis was conducted to compare men and women on individual scores at baseline in order to identify baseline differences. Subsequently, an analysis of covariance (ANCOVA) was performed to examine gender differences in symptom outcomes at 24 M.

The ANCOVA included the variables that showed a significant baseline difference between men and women, as well as levodopa dosage at 24 M was covariates, in order to correct for different levodopa doses taken at follow-up.

Results were expressed as mean ± standard deviation (SD), along with the corresponding p-value. Statistical significance was set at α < 0.05.

## Results

Two hundred and eighty-nine patients (174 men and 115 women) underwent baseline examination between February 2019 and November 2022. Four patients died during the study (2 men and 2 women). Causes of death were: metastatic cancer (not known at baseline), COVID-19 pneumonia, cardiac arrest, undefined for one patient. Sixty-nine dropouts (33 men, 36 women) were ascribed to the restrictions during the COVID-19 pandemic, preventing many patients from returning for the study assessments. After excluding drop-outs, a total of 216 patients, 139 (79.9%) men and 77 (67%) women, completed the 24-month follow-up and, thus, represent the study cohort. As shown in Table 1, no significant differences were observed between the two groups in terms of age (*p* = 0.130), disease duration (*p* = 0.981), or medication dosage at baseline (*p* = 0.381). Levodopa was started at baseline visit. At the 24 M follow-up daily doses of Levodopa were slightly higher in men as compared to women (mean ± SD: 327.64 ± 148.02 vs 286.69 ± 125.63, p = 0.016; Table 1). At baseline, 54.6% of men and 46,1% of women were treated with at least one antiparkinsonian drug. Rasagiline was the most prescribed medication, taken in 28.7% of men and 22.6% of women; dopamine agonists were taken by 36% of men and 33% of women. LEDD excluding levodopa at baseline and 2y follow-up was similar in men and women (Table 1).

**Table 1:** Clinical and demographic features of the enrolled cohort

Variables	Men (*n =* 139) Mean ± SD	Women (*n =* 77) Mean ± SD	p-value
Age (years)	63.64 ± 9.09	65.64 ± 9.32	0.130
Disease Duration (months)	24.55 ± 23.15	24.21 ± 23.98	0.981
LEDD at Baseline (mg/die)	123.88 ± 128.78	107.92 ± 127.14	0.381
LEDD (without Levodopa) at 24 M (mg/die)	139.66 ± 138.22	114.95 ± 121.84	0.191
Levodopa at 24 M (mg/die)	327.64 ± 148.02	286.69 ± 125.63	**0.016**

Mean ± SD for clinical-demographic variables and corresponding p-values from univariate statistical analysis

Significant differences are highlighted in bold

*LEDD* levodopa equivalent daily dose; *M* months; *Mg/die* milligrams per day; *N* number; *SD* standard deviation

At baseline (Table 2), women reported higher cardiovascular (mean ± SD: 10.04 ± 3.10 vs 9.67 ± 2.45, *p* = 0.006) and thermoregulatory (mean ± SD: 5.97 ± 2.22, vs 5.29 ± 1.84, *p* = 0.025) dysfunction compared to men on the SCOPA-AUT scale, while men had lower attention/memory scores (mean ± SD: 1.50 ± 2.84 vs 2.84 ± 4.76, *p* = 0.040) and higher sexual dysfunction (mean ± SD: 2.38 ± 3.88 vs 1.13 ± 2.94, *p* = 0.017) on the NMSS. Moreover, cardiovascular symptoms showed a statistical trend toward higher scores in women (mean ± SD: 0.84 ± 1.50 vs 0.43 ± 0.93, *p* = 0.052) on the NMSS. As for the UPDRS-Part I, women scored significantly higher than men on anxious mood (mean ± SD: 1.23 ± 0.97 vs 0.77 ± 0.92, p < 0.001), pain and other sensations (mean ± SD: 0.82 ± 0.91 vs 0.57 ± 0.77, *p* = 0.032), and fatigue (mean ± SD: 0.84 ± 0.80 vs 1.22 ± 0.99, *p* = 0.006), while presenting lower daytime sleepiness (mean ± SD: 0.60 ± 0.80 vs 0.98 ± 0.89, *p* = 0.002). Regarding quality of life, women reported higher impact on mobility (mean ± SD: 8.03 ± 7.53 vs 4.06 ± 5.01, p < 0.001), emotional well-being (mean ± SD: 6.56 ± 4.45 vs 3.96 ± 4.07, p < 0.001), and bodily discomfort (mean ± SD: 3.01 ± 2.54 vs 2.22 ± 1.95, *p* = 0.044) sub-scores of the PDQ-39. As for the MOCA, men performed better than women on attention (mean ± SD: 5.33 ± 1.39 vs 4.83 ± 1.58, *p* = 0.003). Mean baseline score of the HAM-D was significantly higher in women than men (mean ± SD: 7.54 ± 4.63 vs 5.50 ± 4.84, p = 0.003). Finally, women presented higher scores than men in anxiety (mean ± SD: 3.10 ± 2.58 vs 2.24 ± 1.99, *p* = 0.001), weight concerns (mean ± SD: 0.23 ± 0.54 vs 0.11 ± 0.40, *p* = 0.016), and cognitive disturbance (mean ± SD: 0.71 ± 1.23 vs 0.57 ± 1.07, *p* = 0.029), as assessed by the HAM-A scale.

**Table 2:** Baseline differences in NMSs scores between genders

NMSs	Men Mean ± SD	Women Mean ± SD	p-value
SCOPA-AUT			
Gastrointestinal	9.67 ± 2.45	10.04 ± 3.10	0.703
Urinary	9.64 ± 2.71	9.59 ± 2.76	0.763
Cardiovascular	3.36 ± 0.73	3.78 ± 1.26	**0.006**
Thermoregulatory	5.29 ± 1.84	5.97 ± 2.22	**0.025**
Pupillomotor	1.38 ± 0.67	1.60 ± 1.01	0.289
NMSS			
Cardiovascular	0.43 ± 0.93	0.84 ± 1.50	0.052
Sleep/fatigue	3.69 ± 4.99	5.22 ± 5.64	0.135
Mood/Cognition	5.23 ± 10.14	8.04 ± 14.18	0.119
Perceptual problems/hallucinations	0.04 ± 0.25	0.24 ± 0.80	0.083
Attention/Memory	1.50 ± 2.84	2.84 ± 4.76	**0.040**
Gastrointestinal tract	2.87 ± 4.15	2.60 ± 3.75	0.596
Urinary	5.12 ± 6.28	5.30 ± 7.72	0.445
Sexual functions	2.38 ± 3.88	1.13 ± 2.94	**0.017**
Miscellaneous	2.70 ± 3.93	4.10 ± 5.96	0.271
QUIP			
Game	0.05 ± 0.42	0.01 ± 0.11	0.915
Hypersexuality	1.09 ± 2.30	0.94 ± 2.00	0.620
Compulsive buying	0.89 ± 1.89	1.01 ± 2.05	0.892
Compulsive eating	1.19 ± 2.72	1.29 ± 2.32	0.176
Hobby	1.20 ± 2.18	1.21 ± 2.03	0.878
Pathological Gambling	0.33 ± 1.17	0.50 ± 1.51	0.268
Dopamine dysregulation syndrome	0.49 ± 1.67	0.55 ± 1.65	0.250
UPDRS -Part I			
Cognitive Impairment	0.31 ± 0.53	0.37 ± 0.63	0.719
Hallucinations and Psychosis	0.02 ± 0.15	0.05 ± 0.27	0.474
Depressed Mood	0.73 ± 0.86	0.90 ± 0.99	0.263
Anxious Mood	0.77 ± 0.92	1.23 ± 0.97	** < 0.001**
Apathy	0.50 ± 0.75	0.42 ± 0.80	0.251
Characteristics of Dopamine Dysregulation Syndrome	0.12 ± 0.49	0.14 ± 0.42	0.327
Sleep Disorders	0.86 ± 1.02	0.83 ± 1.05	0.753
Daytime Sleepiness	0.98 ± 0.89	0.60 ± 0.80	**0.002**
Pain and Other Sensations	0.57 ± 0.77	0.82 ± 0.91	**0.032**
Urinary Problems	0.72 ± 0.86	0.76 ± 1.03	0.716
Constipation Problems	0.76 ± 0.96	0.81 ± 0.99	0.806
Feeling of Fainting When Assuming an Upright Position	0.35 ± 0.61	0.36 ± 0.64	0.924
Fatigue	0.84 ± 0.80	1.22 ± 0.99	**0.006**
PDQ −39			
Mobility	4.06 ± 5.01	8.03 ± 7.53	** < 0.001**
Activity of Daily Living	3.49 ± 3.06	4.33 ± 3.99	0.291
Emotional well-being	3.96 ± 4.07	6.56 ± 4.45	** < 0.001**
Stigma	2.48 ± 3.45	2.83 ± 3.58	0.326
Social support	0.67 ± 1.67	0.88 ± 1.71	0.247
Cognition	1.94 ± 2.14	2.47 ± 2.80	0.266
Communication	0.97 ± 1.41	0.94 ± 1.54	0.397
Bodily discomfort	2.22 ± 1.95	3.01 ± 2.54	**0.044**
AES			
Behavioral apathy	8.25 ± 2.40	8.06 ± 2.66	0.682
Cognitive apathy	14.33 ± 4.03	13.85 ± 4.42	0.429
Emotional apathy	3.79 ± 1.19	3.83 ± 1.51	0.865
Other	5.59 ± 1.76	5.62 ± 2.21	0.947
MoCA			
Language	5.06 ± 1.42	4.90 ± 1.49	0.324
Attention	5.33 ± 1.39	4.83 ± 1.58	**0.003**
Visuospatial	5.41 ± 1.84	4.82 ± 2.31	0.149
Orientation	7.61 ± 2.35	7.90 ± 2.28	0.246
HAM-A			
Anxiety	2.24 ± 1.99	3.10 ± 2.58	**0.001**
Weight	0.11 ± 0.40	0.23 ± 0.54	**0.016**
Cognitive Disturbance	0.57 ± 1.07	0.71 ± 1.23	**0.029**
Diurnal Variation	0.62 ± 1.18	0.61 ± 1.05	0.627
Slowness	2.07 ± 1.80	2.30 ± 2.05	0.529
Sleep	1.08 ± 1.43	1.21 ± 1.58	0.589
HAM-D	5.50 ± 4.84	7.54 ± 4.63	**0.003**

Mean ± SD and corresponding p-values from univariate statistical analysis. Statistical significance was set at α < 0.05

Significant differences are highlighted in bold

*AES* apathy evaluation scale; *HAM-A* hamilton anxiety rating scale; *HAM-D*, hamilton depression rating scale; *MDS-UPDRS* movement disorder society–unified Parkinson’s disease rating scale; *MoCA*, montreal cognitive assessment; *NMSs* non-motor symptoms; *NMSS* non-motor symptoms severity scale; *PsDQ-39* 39-item Parkinson’s disease quality of life questionnaire; *QUIP* questionnaire for impulsive-compulsive disorders in Parkinson’s disease; *SCOPA-AUT* scale for outcomes in Parkinson’s disease – Autonomic; *SD* standard deviation

Table 3 presents the results of the ANCOVA analysis evaluating outcomes at 24 months corrected for the baseline scores and the levodopa dose at 24 M.

**Table 3:** Results of the ANCOVA analysis evaluating outcomes at 24 months corrected for the baseline scores and the levodopa dose at 24 M

NMSs	Men Mean ± SD	Women Mean ± SD	*p*-value
SCOPA-AUT			
Gastrointestinal	10.10 ± 2.88	9.60 ± 2.93	0.096
Urinary	9.92 ± 2.73	9.28 ± 3.10	0.205
Cardiovascular	3.48 ± 0.82	3.38 ± 0.98	0.132
Thermoregulatory	5.16 ± 1.63	5.60 ± 2.20	0.195
Pupillomotor	1.30 ± 0.62	1.50 ± 0.82	0.175
NMSS			
Cardiovascular	0.53 ± 1.19	0.83 ± 1.76	0.396
Sleep/fatigue	3.67 ± 4.47	3.78 ± 3.39	0.851
Mood/Cognition	4.57 ± 9.28	4.29 ± 6.64	0.078
Perceptual problems/hallucinations	0.22 ± 0.95	0.60 ± 2.44	0.811
Attention/Memory	1.85 ± 4.60	2.42 ± 4.15	0.969
Gastrointestinal tract	3.33 ± 4.88	2.26 ± 3.31	0.362
Urinary	4.78 ± 6.42	5.49 ± 7.95	0.455
Sexual functions	2.01 ± 4.46	1.68 ± 5.50	0.823
Miscellaneous	2.08 ± 3.64	2.30 ± 3.23	0.818
QUIP			
Game	0.18 ± 1.23	0.10 ± 0.62	0.498
Hypersexuality	1.88 ± 2.70	0.50 ± 1.22	** < 0.001**
Compulsive buying	0.47 ± 1.30	0.56 ± 1.53	0.724
Compulsive eating	1.63 ± 2.70	1.87 ± 3.51	0.528
Hobby	0.62 ± 1.63	0.92 ± 2.11	0.199
Pathological Gambling	0.57 ± 1.37	0.58 ± 1.74	0.912
Dopamine dysregulation syndrome	0.50 ± 1.35	0.41 ± 1.49	0.502
UPDRS -Part I			
Cognitive Impairment	0.29 ± 0.62	0.55 ± 0.76	**0.016**
Hallucinations and Psychosis	0.09 ± 0.39	0.17 ± 0.60	0.237
Depressed Mood	0.55 ± 0.85	0.76 ± 0.91	0.137
Anxious Mood	0.61 ± 0.77	1.03 ± 0.93	**0.015**
Apathy	0.45 ± 0.75	0.37 ± 0.69	0.868
Characteristics of Dopamine Dysregulation Syndrome	0.12 ± 0.40	0.12 ± 0.43	0.951
Sleep Disorders	0.99 ± 1.12	0.96 ± 1.09	0.996
Daytime Sleepiness	0.85 ± 0.82	0.69 ± 0.79	0.527
Pain and Other Sensations	0.58 ± 0.76	0.86 ± 0.88	**0.012**
Urinary Problems	0.84 ± 0.89	0.84 ± 0.97	0.903
Constipation Problems	0.79 ± 0.99	0.76 ± 0.90	0.744
Feeling of Fainting When Assuming an Upright Position	0.31 ± 0.55	0.49 ± 0.81	**0.044**
Fatigue	0.77 ± 0.82	1.01 ± 0.85	0.307
PDQ-39			
Mobility	4.17 ± 6.15	8.26 ± 9.36	**0.009**
Activity of Daily Living	3.38 ± 3.52	3.28 ± 4.11	0.370
Emotional well-being	3.42 ± 3.51	5.79 ± 4.75	**0.014**
Stigma	1.81 ± 2.88	2.10 ± 3.04	0.708
Social support	0.56 ± 1.39	0.78 ± 1.33	0.379
Cognition	2.33 ± 2.23	2.88 ± 2.27	0.296
Communication	1.10 ± 1.52	0.97 ± 1.82	0.644
Bodily discomfort	2.27 ± 2.17	2.73 ± 2.23	0.536
AES			
Behavioral apathy	8.36 ± 2.45	7.91 ± 2.79	0.279
Cognitive apathy	14.20 ± 4.46	13.78 ± 4.69	0.834
Emotional apathy	3.79 ± 1.29	3.74 ± 1.35	0.757
Other	5.37 ± 1.86	5.42 ± 1.86	0.182
MoCA			
Language	5.03 ± 1.46	4.62 ± 1.98	0.182
Attention	5.14 ± 1.61	4.46 ± 2.02	0.136
Visuospatial	5.43 ± 2.06	4.40 ± 2.60	**0.022**
Orientation	7.83 ± 2.57	7.60 ± 3.08	0.363
HAM-A			
Anxiety	1.83 ± 2.13	2.83 ± 2.69	**0.030**
Weight	0.06 ± 0.26	0.04 ± 0.25	0.428
Cognitive Disturbance	0.54 ± 1.06	0.78 ± 1.65	0.544
Diurnal Variation	0.60 ± 1.17	0.56 ± 1.06	0.793
Slowness	1.51 ± 1.98	1.68 ± 1.87	0.643
Sleep	1.00 ± 1.29	1.18 ± 1.63	0.446
HAM-D	5.80 ± 5.49	6.78 ± 6.24	0.528

Statistical significance was set at α < 0.05

Significant differences are highlighted in bold

*AES* apathy evaluation scale; *HAM-A* hamilton anxiety rating scale; *HAM-D* hamilton depression rating scale; *M* months; *MDS-UPDRS* movement disorder society–unified Parkinson’s disease rating scale; *MoCA* montreal cognitive assessment; *NMSs* non-motor symptoms; *NMSS* non-motor symptoms severity scale; *PDQ-39* 39-item Parkinson’s disease quality of life questionnaire; *QUIP* questionnaire for impulsive-compulsive disorders in Parkinson’s disease; *SCOPA-AUT* scale for outcomes in Parkinson’s disease – autonomic; *SD* standard deviation

Significant gender differences were found in the hypersexuality subscale of the QUIP, with higher mean ± SD scores in men compared with women (mean ± SD: 1.88 ± 2.70 vs 0.50 ± 1.22, p < 0.001) (Table 3). Significant differences were also observed in UPDRS Part I, with higher mean scores in women than men, specifically in the cognitive impairment (mean ± SD: 0.55 ± 0.76 vs 0.29 ± 0.62, *p* = 0.016), anxious mood (mean ± SD: 1.03 ± 0.93 vs 0.61 ± 0.77, *p* = 0.015), pain and other sensations (mean ± SD: 0.86 ± 0.88 vs 0.58 ± 0.76, *p* = 0.012), and feelings of fainting when assuming an upright position (mean ± SD: 0.49 ± 0.81 vs 0.31 ± 0.55, *p* = 0.044) items. Regarding the PDQ-39, significant differences emerged in the mobility (mean ± SD: 8.26 ± 9.36 vs 4.17 ± 6.15, *p* = 0.009) and emotional well-being (mean ± SD: 5.79 ± 4.75 vs 3.42 ± 3.51, *p* = 0.014) subdomains, with higher mean scores in women compared with men. A significant difference was also found in the visuospatial subdomain of the MoCA (mean ± SD: 5.43 ± 2.06 vs 4.40 ± 2.60, *p* = 0.022), with worse performance in women. Finally, a significant gender difference was observed in the anxiety item of the HAM-A (mean ± SD: 2.83 ± 2.69 vs 1.83 ± 2.13, *p* = 0.030), with women showing higher scores than men (see Table 3).

## Discussion

In our multi-center longitudinal study, we analyzed the role of gender in NMSs changes over a 2-year follow-up period in a large sample of early stage-levodopa-naïve PD patients. This is the first study comprehensively investigating the whole range of NMSs in such a population.

At baseline, men showed significantly higher sexual dysfunction and daytime sleepiness than women, consistently with previous literature [13, 15–18]. On the other hand, in our cohort women showed worse symptoms than men regarding cardiovascular and thermoregulatory symptoms, anxiety, pain, mobility, emotional wellbeing, and bodily discomfort domains of quality of life. Our data are partially consistent with previous studies. Solla et al. [16], in a cross-sectional study conducted on a smaller sample of patients already treated with levodopa and dopamine agonists, showed that women had higher scores in cardiovascular and mood domains than men. Other cross-sectional studies, enrolling levodopa-treated PD patients with longer disease duration as compared to our cohort, found that anxiety, sadness, depression and pain are more common in women than men affected by PD [13, 17, 18]. In our study high pain severity was reported more commonly in women than men with PD, according to previous cross-sectional, observational studies on large samples of levodopa-treated patients [8, 19]. Moreover, at baseline women had higher scores than men on HAM-D, which assesses multiple facets of the depressive construct, including depressed mood, feelings of guilt, suicidal ideation, insomnia (early, middle and late), psychomotor changes, anhedonia, psychic and somatic anxiety, and a range of somatic and neurovegetative symptoms.

Consistently, a large meta-analysis of 240 observational, mostly cross-sectional studies, aiming at describing sex differences in neuropsychiatric symptoms across alpha-synucleinopathies, confirmed that female sex was associated with a higher prevalence of anxiety and depression [20]. Indeed, depression and anxiety disorders are more common among women than men (5.1% vs 3.6%; 4.6% vs 2.6% worldwide, respectively) also in the general population according to the WHO [21]. The mechanisms leading to the higher prevalence of depression and anxiety in women are not completely understood. The female predisposition to depression is thought to involve different biological processes, such as genetic vulnerability, hormonal fluctuations linked to reproductive function and psychosocial factors. e.g. social status, role-stress, victimization, and coping style [22].

As for quality of life, our baseline results showed a poorer quality of life in women with PD than men, especially in terms of emotional well-being, social support and psychological well-being, consistently with some previous studies [7, 23–26]. Indeed, depression and fatigue are reported as the main causes of poorer health-related quality of life in women, even in the early stages of PD [27].

After adjusting for baseline variables and levodopa dosage, we found that some gender differences were confirmed at 2-year follow-up, whereas other differences emerged as relevant only at follow-up. We found gender differences in our sample both at baseline and follow-up regarding: anxious mood, pain and other sensations assessed with the UPDRS-Part I, mobility and emotional well-being assessed with the PDQ-39, anxiety assessed with the HAM-A. These findings indicate that such gender differences are not influenced by disease duration or the initiation of levodopa therapy.

On the other hand, some gender differences were evident only at follow-up, with women showing significantly greater impairment than men in anxious mood, pain, emotional well-being, mobility, and light-headedness. Cognitive decline, as measured by UPDRS Part I, as well as visuospatial deficits assessed through the MoCA, were also more pronounced in women at follow-up. Finally, at follow-up hypersexuality domain of the QUIP showed higher scores in men than in women, indicating a more severe impulse control disorder over time in men particularly affecting this specific domain. We suggest that either disease progression or change in dopaminergic treatment may account for such differences emerging at 2-year follow-up.

Baseline differences in cardiovascular and thermoregulatory symptoms, sexual function, daytime sleepiness, fatigue, bodily discomfort and attention disappeared at follow-up, suggesting that disease duration or levodopa treatment may affect such differences, with men and women showing similar progression over time in spite of initial gender differences.

Longitudinal evidence describing how non-motor symptom trajectories differ between men and women, particularly in the early stages of the disease and in relation to the initiation of dopaminergic therapy, remains limited. To date, there are no multicenter longitudinal studies evaluating whether, and to what extent, gender influences changes in NMSs profiles among levodopa-naïve patients starting dopaminergic treatment. Only a few studies addressed this topic, although with different designs, often privileging methodological complexity and trajectory modeling over a clearly delineated clinical question [28–31]. With our data, we sought to fill this gap by addressing a clinically specific question, namely, the evaluation of gender effects at a clinically meaningful time point, using a focused and rigorous analytic approach, and ultimately to promote personalized management of NMSs in early PD.

A closer look at the literature indicates that increased sexual drive is consistently reported in men as compared with women in previous studies [28, 32]. For example, Marković et al. [29], in a prospective study of 106 patients with early PD treated with levodopa and/or other dopaminergic drugs, reported that men had a 14-fold higher risk of presenting or developing hypersexuality over time, showing that the prevalence of ICDs increased from 19.8% at baseline to 29.2% at 5-year follow-up, with a significant rise already detectable at the 2-year follow-up. The 2-year prospective study by Picillo et al. [28], conducted in drug-naïve PD patients, also reported higher hypersexuality in men, with women showing a worse progression as regards anxiety, depressed mood, pain, fatigue, and emotional well-being. These findings partially align with our results, with some discrepancies probably reflecting different scales and statistical design used in the previous paper.

Regarding mood-related variables, a previous study longitudinally assessing a small cohort of drug-naïve PD patients over a 4-year follow-up, after co-variating for levodopa dose, also confirmed that pain, anxiety, and mood alterations were more frequently reported by women [33].

In a longitudinal study by Santos García et al. [34], following a large cohort of levodopa-treated PD patients with 24 months of follow-up, apathy, pain, and the physical component of the Visual Analogue Fatigue Scale worsened more in women than men, in line with our results.

As for gender differences in the cognitive domain, generally women show a lower risk of developing cognitive impairments compared with male patients [35]. More specifically, Cholerton, et al. [11] found that the primary predictive factor in the transition from no cognitive impairment to PD-MCI or dementia was male sex. Our data regarding cognitive domain in early disease stages of PD are consistent with results by Locascio et al. [36]. In fact, in a 10-year prospective longitudinal study including 97 PD patients treated with levodopa and 41 controls, they found a greater longitudinal decline in visuospatial functioning in women as compared to men. Similarly, Bakeberg et al. [37], in a longitudinal study using the Addenbrooke’s Cognitive Examination in a cohort of 392 treated patients with PD, reported that women exhibited greater longitudinal deterioration in visuospatial abilities, as well as in attention/orientation and memory domains, whereas men showed a more pronounced decline in global cognitive performance and language.

Finally, we must acknowledge that the Parkinson’s Progression Markers Initiative (PPMI) has provided important insights into early NMSs changes and sex differences [29, 30]. However, papers derived from the PPMI cohort primarily focus on modeling symptom trajectories, whereas our study addresses a more specific and clinically grounded question. For example, Simuni et al. [29], analyzing 423 levodopa-naïve PD subjects and 196 controls followed for 2 years, showed that NMSs burden increases during the first two years of disease and identified female sex as an independent predictor of greater NMSs severity at baseline. While this is partially consistent with our findings from an overall perspective, their methodology differed since they employed statistical models optimized for characterizing longitudinal trajectories and did not describe specific NMSs progression across genders. Conversely, our analysis was specifically designed to assess whether end-point differences at 24 months were independent of baseline values and treatment exposure, making our approach particularly suitable for isolating sex-specific effects at a clinically relevant time point.

On the opposite end of the spectrum compared with our results are the findings by Picillo et al. [30]. Although both studies examine gender-related progression of non-motor symptoms, substantial methodological and clinical differences help contextualize the discrepancies. Picillo et al. [30], analyzing 423 untreated PD patients from the PPMI followed over 5 years, used generalized linear models with gender × time interaction terms to evaluate differences in symptom trajectories. They found no significant gender × time interaction for most NMSs domains (including dysautonomia, anxiety, sleep, depression, and ICDs), suggesting similar progression rates in men and women; however, they did observe a greater longitudinal worsening in men in the total MDS-UPDRS Part I score. In contrast, gender differences in our cohort emerged at the 24-month follow-up and remained significant after adjustment for levodopa, suggesting that sex-related disparities may manifest as persistent end-point differences rather than divergent longitudinal trajectories. From this perspective, the null interaction effects reported by Picillo et al. [30] may mask clinically relevant absolute differences that become evident when focusing on follow-up outcomes rather than on slope-based comparisons. Additionally, the longer follow-up (5 years vs 2 years) in the previous study may have attenuated early differences, whereas our study captures differences emerging soon after the initiation of levodopa. Our approach, highlighting gender-specific differences at a clinically relevant time point, allows us to identify distinctions that may be attenuated or undetectable in trajectory-oriented statistical models.

We must recognize that our study has some limitations. At baseline patients were levodopa-naïve but not completely drug-naïve, since about 50% of both men and women were treated with at least one antiparkinsonian drug. Moreover, the 24-month follow-up period might limit full insight into long-term NMS trajectories in early PD. Further studies with a longer monitoring are warranted in early drug-naïve PD to better capture progression patterns according to gender. Finally, we recognize that objective evaluations, such as olfactory testing, could have strengthened our findings. However, we underline that NMS evaluation over time was performed by means of a very extensive battery of scales specifically designed or validated to assess NMSs in PD population.

In conclusion, our multicenter longitudinal study provides new evidence on the significant role of gender in the expression and early progression of non-motor symptoms in levodopa-naïve PD patients followed for the first two years of levodopa treatment. By analyzing a broad range of NMSs domains using multiple dedicated scales and adjusting for both baseline and levodopa dosage differences, we demonstrated that several gender differences in NMSs were present at baseline and persisted independently from disease duration and levodopa start, while other gender differences appeared over time (pain, anxiety, emotional well-being, hypersexuality). We suggest that our study, thanks to a comprehensive assessment of non-motor symptoms, also including subscore/subdomain analysis, clarified aspects not captured by broader longitudinal trajectory models, offering useful insights for personalized early management of NMSs in PD.

Overall, our data support the existence of distinct phenotypic profiles between men and women in the early stages of PD and highlight the importance of integrating gender considerations into the assessment and management of non-motor symptoms from the earliest stages of the disease. Our work reinforces the need for personalized treatment approaches and suggests that future longitudinal studies should integrate biological, neuropsychological, and treatment-related aspects to further elucidate the mechanisms underlying gender differences in PD.

## Supplementary Information

Below is the link to the electronic supplementary material.

Supplementary file1 (DOCX 12 KB)

## Data Availability

The data that support the findings of this study are available on request from the corresponding author. The data are not publicly available due to privacy or ethical restrictions.
