# Supplementary material for: Gender differences in non-motor symptoms in Parkinson’s disease: a multicenter longitudinal study
Source: Neurol Sci. 2026 Apr 16;47(5):427. doi: 10.1007/s10072-026-09031-2 (PMC13086900; doi:10.1007/s10072-026-09031-2)
Supplement: Supplementary file 1 — Supplementary file1 (DOCX 12 KB) [file 10072_2026_9031_MOESM1_ESM.docx]

**Supplemental material**

S1. Weintraub D, & Claassen DO (2017). Impulse Control and Related Disorders in Parkinson's Disease. *International review of neurobiology*, *133*, 679–717. https://doi.org/10.1016/bs.irn.2017.04.006.

S2. Hamilton M (1967). Development of a rating scale for primary depressive illness. *The British journal of social and clinical psychology*, *6*(4), 278–296. https://doi.org/10.1111/j.2044-8260.1967.tb00530.x

S3. Hamilton M (1959). The assessment of anxiety states by rating. *The British journal of medical psychology*, *32*(1), 50–55. https://doi.org/10.1111/j.2044-8341.1959.tb00467.x.

S4. Marin RS1. Biedrzycki RC. Firinciogullari S. Reliability and validity of the Apathy Evaluation Scale. Psychiatry Res 1991;38:143-162. doi: 10.1016/0165-1781(91)90040-v.

S5. Marin RS, Biedrzycki RC, & Firinciogullari S (1991). Reliability and validity of the Apathy Evaluation Scale. *Psychiatry research*, *38*(2), 143–162. https://doi.org/10.1016/0165-1781(91)90040-v

S6. Visser M, Marinus J, Stiggelbout AM, & Van Hilten, JJ (2004). Assessment of autonomic dysfunction in Parkinson's disease: the SCOPA-AUT. *Movement disorders : official journal of the Movement Disorder Society*, *19*(11), 1306–1312. https://doi.org/10.1002/mds.20153.

S7. Nasreddine ZS. Phillips NA. Bédirian V. et al. (2005)The Montreal Cognitive Assessment. MoCA: a brief screening tool for mild cognitive impairment. J Am Geriatr Soc, 53:695-699. doi: 10.1111/j.1532-5415.2005.53221.x.

S8. Peto V, Jenkinson C, Fitzpatrick R, Greenhall R (1995) The development and validation of a short measure of functioning and well being for individuals with Parkinson's disease. Qual Life Res, 4:241-248. doi: 10.1007/BF02260863.

S9 Carrozzino D, Patierno C, Fava GA et al. (2020) Rating Scales for Depression: A Critical Review of Clinimetric Properties of Different Versions. *Psychother Psychosom*., 89(3):133–150. doi: 10.1159/000506879.
